# The Roles of Autophagy, Mitophagy, and the Akt/mTOR Pathway in the Pathogenesis of Chronic Rhinosinusitis with Nasal Polyps

**DOI:** 10.1155/2022/2273121

**Published:** 2022-06-14

**Authors:** Chen Wang, Min-Li Zhou, Yong-Cai Liu, Ke-Jia Cheng

**Affiliations:** Department of Otolaryngology, The First Affiliated Hospital, School of Medicine, Zhejiang University, Hangzhou, China

## Abstract

The pathogenesis of CRSwNP is complex and unclear. CRSwNP is subdivided into two types based on the infiltration of EOSs: eCRSwNP and noeCRSwNP. This study was designed to seek the role of autophagy, mitophagy, and Akt/mTOR pathway in these two subtypes of CRSwNP. This study included 29 patients with CRSwNP and 9 controls. The levels of autophagy, mitophagy, and Akt/mTOR pathway-related proteins in nasal tissues were quantified using western blot analysis. Levels of eosinophilic inflammation-related cytokines in nasal tissues were quantified by enzyme-linked immunosorbent assay. Immunohistochemistry was also used to evaluate autophagy, mitophagy, and Akt/mTOR pathway-related protein expression and distribution in nasal polyps and control tissues. Transmission electron microscopy was used to detect the formation of autophagosomes and mitochondrial autophagosomes. Masson's trichrome and periodic acid–Schiff Alcian blue staining were used to evaluate the severity of tissue remodeling. The expression of p-Akt/Akt and p-mTOR/mTOR was upregulated in patients with eCRSwNP or noeCRSwNP. Beclin 1, PINK1, BNIP3, and FUNDC1 levels were significantly reduced in the nasal polyps of patients with eCRSwNP or noeCRSwNP. Autophagosomes and mitochondrial autophagosomes formed less frequently in the nasal polyps of patients with eCRSwNP or noeCRSwNP. Levels of IL-4, IL-5, IL-13, and ECP and the eotaxins CCL11, CCL24, and CCL26 were elevated in the nasal polyps of patients with eCRSwNP or noeCRSwNP. Tissue remodeling is enhanced in patients with eCRSwNP or noeCRSwNP. The Akt/mTOR pathway, eosinophilic inflammation, and tissue remodeling are activated in the nasal polyps of patients with eCRSwNP or noeCRSwNP. The downregulation of autophagy and mitophagy is also observed in eosinophilic and noneosinophilic nasal polyps. The targeting of mitophagy may provide new therapeutic options for different endotypes of CRSwNP.

## 1. Introduction

Chronic rhinosinusitis with nasal polyps (CRSwNP) is a chronic inflammation of the nasal cavity and sinuses, accompanied by the formation of polyps. The pathogenesis of CRSwNP is complex and may involve infection, inflammation, anatomical abnormalities, immunodeficiencies, superantigens, and bacterial biofilms [1]. CRSwNP is subdivided into two types based on the infiltration of eosinophils (EOSs): eosinophilic CRSwNP (eCRSwNP) and noneosinophilic CRSwNP (noeCRSwNP) [2]. These two subtypes of CRSwNP have characteristic pathogeneses, clinical presentations, and prognoses.

Eosinophilic inflammation is an important factor for eCRSwNP. This inflammation stimulates the secretion of interleukin (IL)-4, IL-5, IL-13, eosinophil cationic protein (ECP), and eotaxin. CRSwNP patients also exhibit tissue remodeling [3]. Persistent eosinophilic inflammation and tissue remodeling may result in recurrence of CRSwNP.

Autophagy is a lysosomal-dependent degradation process that maintains cell homeostasis by targeting proteins and organelles [4]. It includes the following stages: extension of a phagocytic bubble, formation of autophagosomes, fusion of autophagosomes and lysosomes, and degradation mediated by lysosomal enzymes [5]. Three forms of autophagy have been identified: macroautophagy, microautophagy, and molecular chaperone-mediated autophagy [6]. Some autophagy-related proteins play critical roles in the various stages of autophagy. For example, Beclin 1 plays a crucial role in the formation of phagocytic bubble bilayers [7]. The binding of microtubule-associated protein kinase light chain 3 (LC3) to the autophagosome bilayer is another key step during autophagy [8]. An autophagy cargo protein, p62, has multiple domains that interact with LC3 and ubiquitin. Because p62 expression is reduced by successful autophagy, p62 labeling is used to track the process [9]. Autophagy can protect cells and tissues by decreasing inflammation, as well as by removing irritants and invading pathogens. However, abnormal autophagy can aggravate tumor development, infections, inflammation, and neurodegenerative and autoimmune diseases [10]. Autophagy can also regulate eosinophilic inflammation and tissue remodeling by modulating fibroblast apoptosis, as well as EOS differentiation, secretion, and apoptosis [11]. Recent research has suggested that autophagy aggravates lower airway inflammation and CRSwNP [12, 13], but these conclusions are controversial [14, 15].

Mitophagy is a form of selective autophagy in which dysfunctional mitochondria are degraded [7]. It prevents the accumulation of reactive oxygen species (ROS), mitochondrial DNA, and mitochondrial antiviral signaling protein, thereby inhibiting inflammation and apoptosis. Mitophagy is regulated by the PTEN-induced putative kinase 1 (PINK1)/parkin-dependent (ubiquitin-dependent) and mitophagy receptor-regulated (nonubiquitin-dependent) pathways [16]. The most important receptors involved in regulating mitophagy are BCL2-interacting protein 3 (BNIP3) and FUN14 domain-containing protein 1 (FUNDC1) [16]. Abnormal mitophagy is associated with neurodegenerative diseases, pulmonary fibrosis, autoimmune diseases, and cancer. In a recent study, decreased levels of mitophagy promoted the proliferation and differentiation of fibroblasts, affecting the pathogenesis of idiopathic pulmonary fibrosis [17]. However, the relationship between mitophagy and eosinophilic inflammation/CRSwNP development remains unclear.

Autophagy and mitophagy are also regulated by the phosphoinositide 3-kinase (PI3K)/protein kinase B (Akt)/mammalian target of rapamycin (mTOR) pathway [18, 19]. Upon phosphorylation by mTOR complex 1, autophagy-related gene (ATG)13 dissociates from ATG1, reducing ATG1 kinase activity and inhibiting autophagy [20]. However, few studies have focused on the relationship between the Akt/mTOR pathway and the development of CRSwNP [21, 22].

Therefore, we investigated the levels of autophagy and mitophagy, activation of the Akt/mTOR pathway, eosinophilic inflammation, and tissue remodeling in patients with each subtype of CRSwNP.

## 2. Materials and Methods

This study was approved by the Research Ethics Committee of the First Affiliated Hospital, College of Medicine, Zhejiang University (Zhejiang, China). The work described has been carried out in accordance with the Code of Ethics of the World Medical Association (Declaration of Helsinki) for experiments involving humans. Informed consent was obtained for experimentation with patients and healthy controls.

### 2.1. Patients

A total of 29 patients with CRSwNP who underwent endoscopic sinus surgery in our hospital were included in this study. CRSwNP was diagnosed in accordance with the guidelines in the European Position Paper on Rhinosinusitis and Nasal Polyps 2020 for the diagnosis and treatment of chronic rhinosinusitis [23]. Nasal tissues from nine patients with no allergic disease who underwent nasal septum corrective surgery or sinus cyst removal were used as control samples.

### 2.2. Hematoxylin and Eosin Staining

We used hematoxylin and eosin (HE) staining (Wuhan Google Biotechnology, Wuhan, China) to detect EOS infiltration. Samples containing more than 10% of cells that exhibited inflammation under 400× high-power field (HPF) microscopy were classified as eCRSwNP samples, and the remaining samples were classified as noeCRSwNP samples.

### 2.3. Western Blot Analysis

The protein levels of phosphorylated Akt (p-Akt), Akt, phosphorylated mTOR (p-mTOR), mTOR, Beclin 1, BNIP3, LC3II/LC3I, FUNDC1, parkin (Abcam, Cambridge, UK), p62, and PINK1 (Affinity Biosciences, Ohio, USA) in nasal tissues were quantified using western blot analysis. Briefly, tissue from each sample was ground, added to lysis solution, incubated on ice for 30 min, and then centrifuged at 12,000 rpm and 4°C for 15 min. A bicinchoninic acid protein assay kit was used to determine protein concentrations. The proteins were resolved using sodium dodecyl sulfate-polyacrylamide gel electrophoresis and electrotransferred to membranes. The membranes were blocked and then incubated overnight at 4°C with a primary antibody. The next day, the membranes were washed five times with TBST buffer and incubated with a secondary antibody at 37°C for 2 h. Then, the membranes were washed five times with TBST buffer. Proteins were visualized using enhanced chemiluminescence.

### 2.4. Enzyme-Linked Immunosorbent Assay

Levels of IL-4, IL-5, IL-13, ECP, eotaxin 1 (CCL11), eotaxin 2 (CCL24), and eotaxin 3 (CCL26) (Mlbio, Shanghai, China) in nasal tissues were quantified in an enzyme-linked immunosorbent assay (ELISA). In brief, the samples (40 *μ*L) were incubated in 96-well plates with the appropriate antibody (10 *μ*L) for 30 min at 37°C. Wash buffer was added to each well for 30 s and then gently tapped out. This procedure was performed five times. Next, 50 *μ*L of horseradish peroxidase conjugate reagent was added to each well, and the plate was incubated and washed as before. Chromogen solution A and chromogen solution B (50 *μ*L each) were added to each well followed by gentle mixing, and the plates were incubated for 15 min at 37°C. Next, 50 *μ*L of stop solution was added to each well, and the optical density at 450 nm was read within 15 min. Protein levels were quantified using a standard curve.

### 2.5. Immunohistochemistry

Immunohistochemistry (IHC) was used to evaluate p-Akt (Servicebio, Wuhan, China), p-mTOR, PINK1, LC3II, p62 (Affinity Biosciences), Beclin 1, BNIP3, and FUNDC1 (Abcam) protein expression and distribution in nasal polyps and control tissues. Briefly, each tissue slice was washed three times in xylene solution for 15 min each time. The slice was then washed twice in anhydrous ethanol solution for 5 min each time, followed by 85% ethanol for 5 min, an alcohol soaking solution for 5 min, and then distilled water. Thereafter, the tissue slice was immersed in citric acid antigenic repair buffer and left to cool. Next, the slice was washed three times in phosphate-buffered saline (PBS) for 5 min each time. The slice was placed in 3% hydrogen peroxide solution and incubated at room temperature for 25 min in the absence of light, after which it was transferred to PBS and washed three times on a decolorizing shaker for 5 min each time. Then, 3% bovine serum albumin was added to cover the tissue evenly, and the slice was sealed at room temperature for 30 min. The blocking fluid was gently removed, the primary antibody was added, and the slice was incubated overnight at 4°C. Next, the slice was transferred to PBS and washed three times on a decolorizing shaker for 5 min each time. The secondary antibody was added, and the slice was incubated at room temperature for 50 min and then transferred to PBS and washed three times for 5 min each time. After the slice was dry, 3,3'-diaminobenzidine solution was added until a brown–yellow color developed, after which the slice was washed in tap water. Next, the slice was restained with Harris hematoxylin for 3 min, washed with tap water, immersed in 1% hydrochloric acid in ethanol for a few seconds, and washed with tap water again. The slice was immersed in ammonia, rinsed with running water, and immersed in ethanol for 5 min. It was washed with 85% ethanol for 5 min and then washed twice in anhydrous ethanol solution for 5 min each time before a final wash in xylene solution for 5 min. The slice was removed from the xylene solution, dried, sealed using neutral gum, and examined using a microscope.

### 2.6. Masson's Trichrome Staining

Masson's trichrome (MT) staining (Wuhan Google Biotechnology) was used to detect collagen, a marker for extracellular matrix components, and assess the extent of tissue remodeling. Briefly, each tissue slice was washed twice in xylene solution for 20 min each time and twice in anhydrous ethanol solution for 5 min each time. The slice was then washed in alcohol soaking solution for 5 min and rinsed with tap water. Subsequently, the slice was soaked overnight in potassium dichromate solution and rinsed with running water. It was then immersed in iron hematoxylin solution for 3 min, rinsed with tap water, immersed in hydrochloric acid in ethanol, and rinsed with tap water again. Next, the slice was immersed in acid fuchsin solution for 5 min, rinsed with running water, and immersed in phosphomolybdic acid solution for 3 min. The slice was immersed in aniline blue dye for 3 min, transferred to 1% glacial acetic acid, and dehydrated by immersion in anhydrous ethanol twice. The slice was immersed in a third solution of anhydrous ethanol for 5 min and transferred to xylene solution for 5 min. Finally, the slice was removed from the xylene solution, sealed using neutral gum, and examined using a microscope. ImageJ software (National Institutes of Health, Bethesda, MD, USA) was used to quantify the area stained blue in three replicates under HPF microscopy.

### 2.7. Periodic Acid–Schiff Alcian Blue Staining

Periodic acid–Schiff Alcian blue (PAS–AB) staining (Wuhan Google Biotechnology) was used to detect mucus produced by goblet cells indicating tissue remodeling. Briefly, each tissue slice was washed twice in xylene solution for 20 min each time and twice in anhydrous ethanol solution for 5 min each time. The slice was then washed with alcohol soaking solution for 5 min and rinsed with tap water. Next, the slice was immersed in Alcian blue dye for 5 min and washed in running water for 2 min. Then, the slice was immersed in periodic acid for 15 min, washed with tap water, and washed twice with distilled water. The slice was immersed in Schiff reagent for 30 min in the absence of light and washed with running water for 5 min. Next, the slice was washed three times in anhydrous ethanol solution for 5 min each time and twice in xylene solution for 5 min each time. Finally, the slice was removed from the xylene solution, sealed using neutral gum, and examined using a microscope. ImageJ software (National Institutes of Health) was used to quantify the areas stained blue and purple/red in three replicates under HPF microscopy.

### 2.8. Transmission Electron Microscopy

The formation of autophagosomes and mitochondrial autophagosomes in nasal polyps and control tissues was observed using transmission electron microscopy (TEM). Briefly, fresh tissue was prepared in a fixator for 2 h at 4°C, rinsed three times with PBS for 15 min each time, and then fixed with 1% osmium/PBS for 2 h at room temperature. The tissue was rinsed three times with PBS for 15 min each time and then dehydrated using successive 15-min washes in increasing concentrations of ethanol (50%, 70%, 80%, 90%, 95%, and 2 × 100%), as well as two washes in 100% acetone. Next, the tissue was treated for 3 h in a 1 : 1 solution of acetone and 812 embedding agent, then overnight in a 2 : 1 solution of acetone and 812 embedding agent, and finally for 6 h in 812 embedding agent alone. The sample was inserted into an embedding plate, which contained 812 embedding agent. This was incubated overnight at 37°C and then polymerized for 48 h at 60°C. Sample sections were stained twice with uranium lead solution for 15 min each time (2% uranium acetate-saturated alcohol, lead citrate solution), dried overnight at room temperature, and examined using TEM.

### 2.9. Statistical Methods

The data were analyzed, and the normality of their distributions was evaluated using SPSS software (ver. 20.0; SPSS, Inc., Chicago, IL, USA). We used *t* tests to analyze normally distributed data and Mann–Whitney *U* tests to analyze nonnormally distributed data. Pearson's test was used to identify correlations between the Akt/mTOR pathway, autophagy, mitophagy, eosinophilic inflammation, and tissue remodeling in normally distributed data. Spearman's test was used to identify correlations between variables with nonnormally distributed data. A *P* value <0.05 was considered to indicate statistical significance.

### 2.10. Ethics Statement

This study was approved by the Research Ethics Committee of the First Affiliated Hospital, College of Medicine, Zhejiang University (Zhejiang, China). The work described has been carried out in accordance with The Code of Ethics of the World Medical Association (Declaration of Helsinki) for experiments involving humans. Informed consent was obtained for experimentation with patients and healthy controls.

## 3. Results

### 3.1. CRSwNP Subtypes

**Figure 1:**
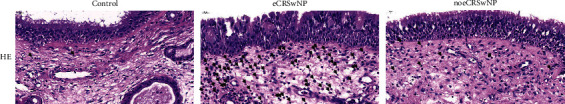
HE staining for nasal tissues of patients with eCRSwNP, noeCRSwNP, or control groups. The quantity of EOSs varied significantly between the eCRSwNP, noeCRSwNP, and control groups. EOSs: black arrows.

Based on the HE staining analyses, 10 patients were assigned to the eCRSwNP group and 19 patients to the noeCRSwNP group. The quantity of EOSs varied significantly between the eCRSwNP, noeCRSwNP, and control groups (Figure 1).

### 3.2. Activation of the Akt/mTOR Pathway in Different CRSwNP Subtypes

**Figure 2:**
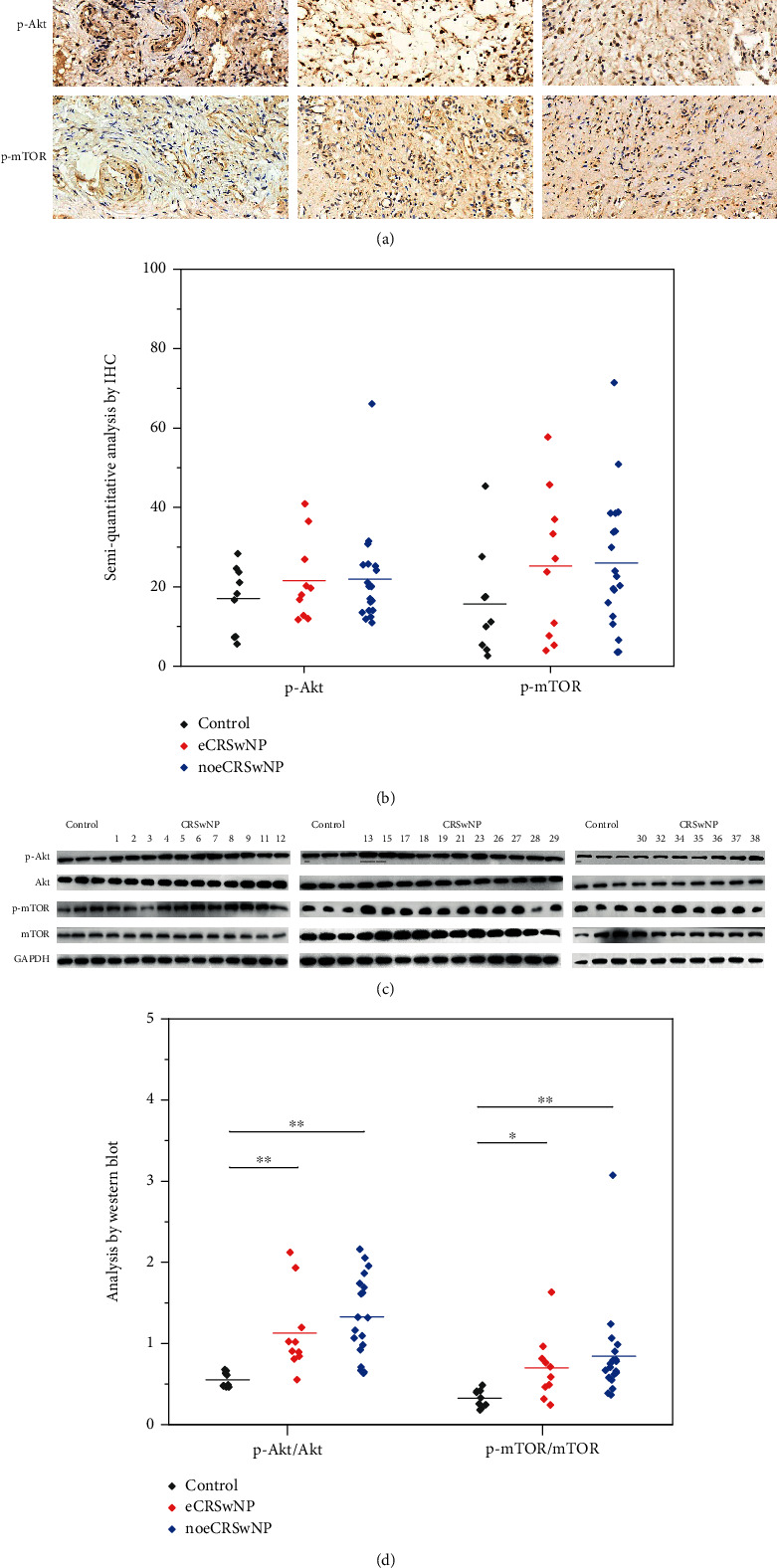
Activation of the Akt/mTOR pathway in different CRSwNP subtypes. (a, b) The IHC analysis showed that the expression levels of p-Akt and p-mTOR in the nasal tissues of patients with eCRSwNP or noeCRSwNP were not significantly different from those observed in control tissues. (c, d) The western blot analysis showed that the expression of p-Akt/Akt and p-mTOR/mTOR was significantly greater in patients with eCRSwNP or noeCRSwNP than in control tissues. ∗*P* < 0.05, ∗∗*P* < 0.001.

The Akt/mTOR pathway plays an important role in various physiological and pathological processes as well as the development of CRSwNP [22]. Our IHC analysis showed that the expression levels of p-Akt and p-mTOR in the nasal tissues of patients with eCRSwNP or noeCRSwNP were not significantly different from those observed in control tissues (Figures 2(a) and 2(b)). Next, western blot analysis was used to quantify the levels of p-Akt, Akt, p-mTOR, and mTOR proteins in nasal tissues. Interestingly, the expression of p-Akt/Akt was significantly greater in patients with eCRSwNP (*P* < 0.001) or noeCRSwNP (*P* < 0.001) than in control tissues (Figures 2(c) and 2(d)). In addition, the expression of p-mTOR/mTOR was also upregulated in patients with eCRSwNP (*P* = 0.015) or noeCRSwNP (*P* < 0.001), compared with control tissues (Figures 2(c) and 2(d)). No significant differences in expression levels were observed between patients with eCRSwNP and those with noeCRSwNP. Increased levels of p-Akt/Akt and p-mTOR/mTOR may indicate the activation of the Akt/mTOR pathway, and our results suggest that the Akt/mTOR pathway is activated in patients with eCRSwNP or noeCRSwNP.

### 3.3. Autophagy in Different CRSwNP Subtypes

**Figure 3:**
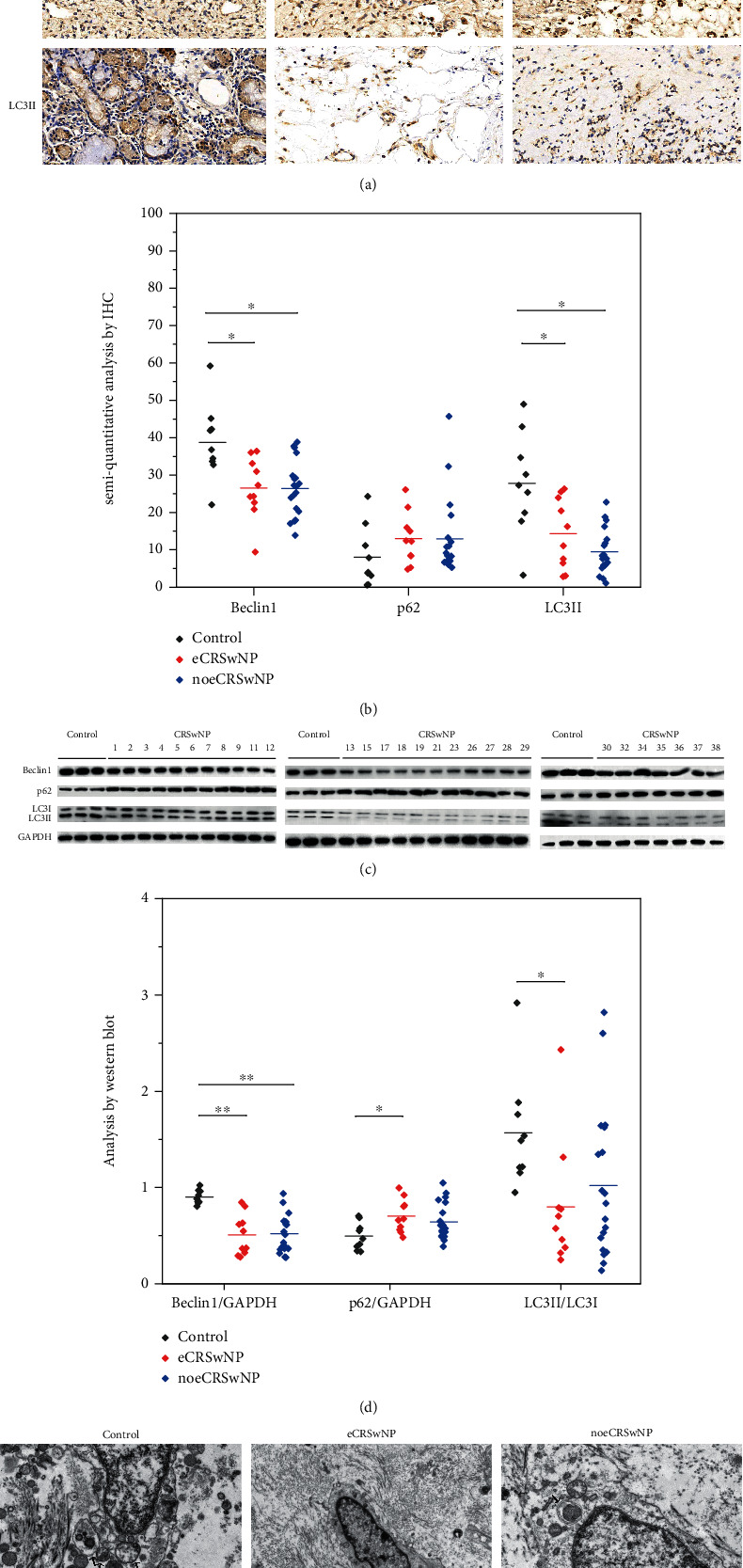
Autophagy levels in different CRSwNP subtypes. **(**a, b) By IHC staining, the number of Beclin 1^+^ and LC3II^+^ cells was significantly reduced in patients with eCRSwNP or noeCRSwNP, compared with control tissues. p62 staining revealed no significant differences among the groups. (c, d) Western blot analysis showed that the levels of Beclin 1 protein in nasal polyps were downregulated in patients with eCRSwNP or noeCRSwNP, compared with control tissues. p62 protein levels increased, whereas LC3II/LC3I levels were significantly reduced, only in the nasal polyps of patients with eCRSwNP, compared with control tissues. (e) TEM showed that autophagosomes formed less frequently in the nasal polyps of patients with eCRSwNP or noeCRSwNP, compared with control tissues. Black arrow: autophagosome. ∗*P* < 0.05, ∗∗*P* < 0.001.

Autophagy plays an important role in many diseases and is involved in CRSwNP pathogenesis [24]. Therefore, we used IHC to detect autophagy-related proteins in nasal tissues. The number of Beclin 1^+^ cells was significantly reduced in patients with eCRSwNP (*P* = 0.01) or noeCRSwNP (*P* = 0.001), compared with control tissues (Figures 3(a) and 3(b)). The number of LC3II^+^ cells was also significantly reduced in patients with eCRSwNP (*P* = 0.022) or noeCRSwNP (*P* = 0.004), compared with control tissues (Figures 3(a) and 3(b)). However, p62 staining revealed no significant differences among the groups (Figures 3(a) and 3(b)). Western blot analysis showed that the levels of Beclin 1 protein in nasal polyps were downregulated in patients with eCRSwNP (*P* < 0.001) or noeCRSwNP (*P* < 0.001), compared with control tissues (Figures 3(c) and 3(d)). Interestingly, p62 protein levels increased (*P* = 0.011), whereas LC3II/LC3I levels were significantly reduced (*P* = 0.006), in the nasal polyps of patients with eCRSwNP, compared with control tissues (Figures 3(c) and 3(d)). To validate these autophagy analyses, we also performed TEM and found that autophagosomes formed less frequently in the nasal polyps of patients with eCRSwNP or noeCRSwNP, compared with control tissues (Figure 3(e)).

Next, we investigated potential associations between the expression of autophagy-related proteins and activation of the Akt/mTOR pathway. However, we found no correlations between the levels of autophagy-related proteins and those of Akt/mTOR pathway proteins.

Together, these data confirm the findings of previous studies and show that autophagy is downregulated in patients with eCRSwNP or noeCRSwNP [25, 26].

### 3.4. Mitophagy in Different CRSwNP Subtypes

**Figure 4:**
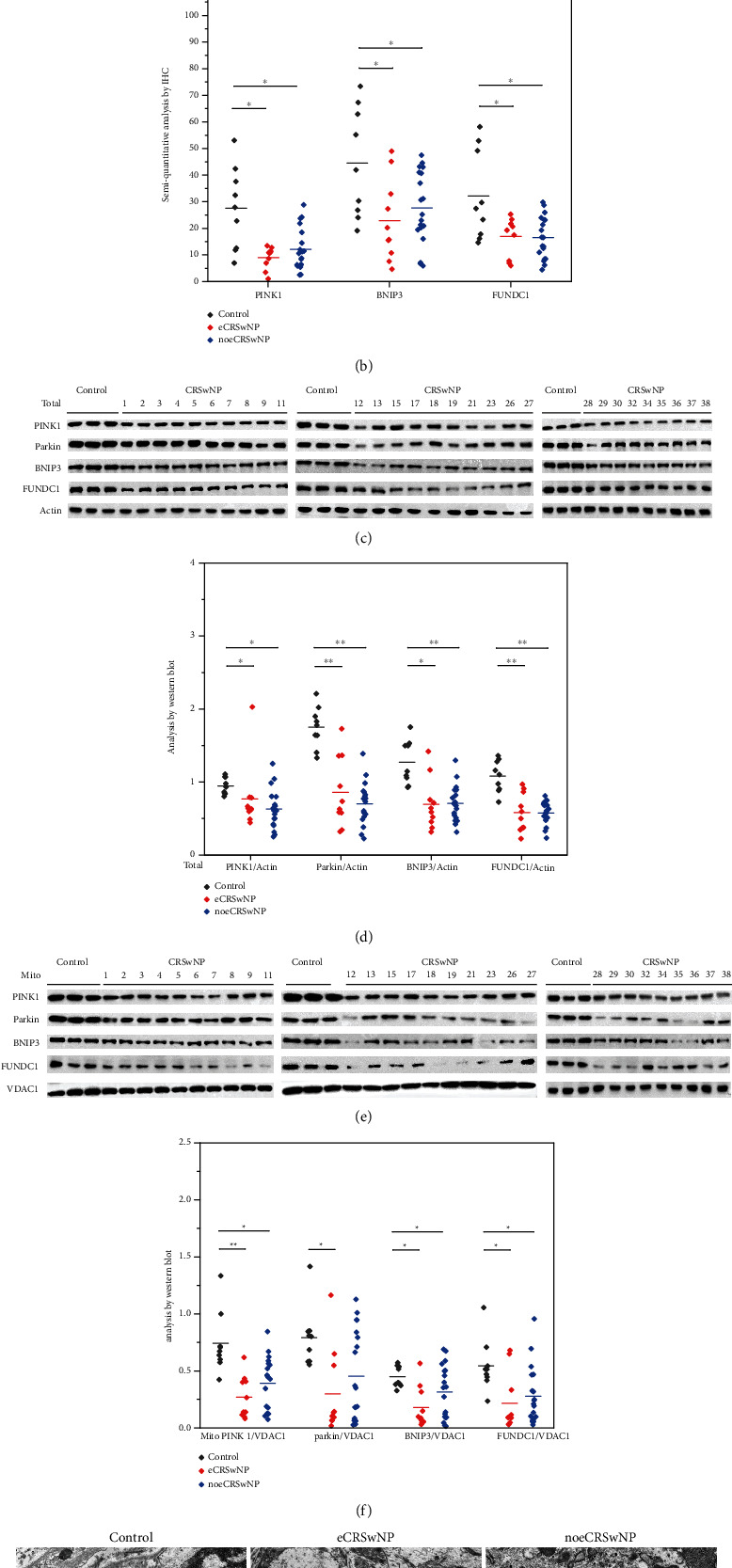
Mitophagy levels in different CRSwNP subtypes. (a, b) The IHC analysis of nasal tissues showed that the expression of PINK1, BNIP3, and FUNDC1 proteins was significantly reduced in patients with eCRSwNP or noeCRSwNP, compared with control tissues. (c, d) Western blot analysis of total proteins revealed reduced levels of PINK1, parkin, BNIP3, and FUNDC1 in the nasal polyps of patients with eCRSwNP or noeCRSwNP, compared with control tissues. (e, f) Western blot analysis of mitochondrial proteins revealed significantly reduced levels of PINK1, parkin, BNIP3, and FUNDC1 in the nasal polyps of patients with eCRSwNP, compared with control tissues. Similar results were found for patients with noeCRSwNP, with the exception of parkin protein levels. (g) TEM showed that mitochondrial autophagosomes formed less frequently in the nasal polyps of patients with eCRSwNP or noeCRSwNP, compared with control tissues. Red arrow: mitochondrial autophagosomes. White arrow: mitochondria. ∗*P* < 0.05, ∗∗*P* < 0.001.

Abnormal mitophagy is associated with various diseases, including neurodegenerative diseases [27], pulmonary fibrosis [28], autoimmune diseases [7], and cancer [29]. However, the role of mitophagy in CRSwNP development remains poorly understood. We investigated mitophagy in patients with CRSwNP and the expression of mitophagy-related proteins in nasal tissues. Our IHC analysis of nasal tissues found that the expression of PINK1, BNIP3, and FUNDC1 proteins was significantly reduced in patients with eCRSwNP (*P* = 0.007, 0.018, and 0.03, respectively) or noeCRSwNP (*P* = 0.018, 0.045, and 0.025, respectively), compared with control tissues (Figures 4(a) and 4(b)). Western blot analysis of total proteins revealed reduced levels of PINK1, parkin, BNIP3, and FUNDC1 in the nasal polyps of patients with eCRSwNP (*P* = 0.002, <0.001, 0.001, and <0.001, respectively) or noeCRSwNP (*P* = 0.002, <0.001, <0.001, and <0.001, respectively), compared with control tissues (Figures 4(c) and 4(d)). Western blot analysis of mitochondrial proteins also revealed significantly reduced levels of PINK1, parkin, BNIP3, and FUNDC1 in the nasal polyps of patients with eCRSwNP (*P* < 0.001, 0.004, 0.002, and 0.013, respectively), compared with control tissues (Figures 4(e) and 4(f)). Similar results were found for patients with noeCRSwNP (PINK1, *P* = 0.001; BNIP3, *P* = 0.04; and FUNDC1, *P* = 0.006), with the exception of parkin protein levels (*P* = 0.076; Figures 4(e) and 4(f)). TEM showed that mitochondrial autophagosomes formed less frequently in the nasal polyps of patients with eCRSwNP or noeCRSwNP, compared with control tissues (Figure 4(g)).

We also investigated potential associations between the expression of mitophagy-related proteins and activation of the Akt/mTOR pathway. However, we found no correlations between the levels of mitophagy-related proteins and those of Akt/mTOR pathway proteins in patients with eCRSwNP or noeCRSwNP.

These results show that mitophagy is downregulated in patients with eCRSwNP or noeCRSwNP.

### 3.5. Cytokines in Different CRSwNP Subtypes

**Figure 5:**
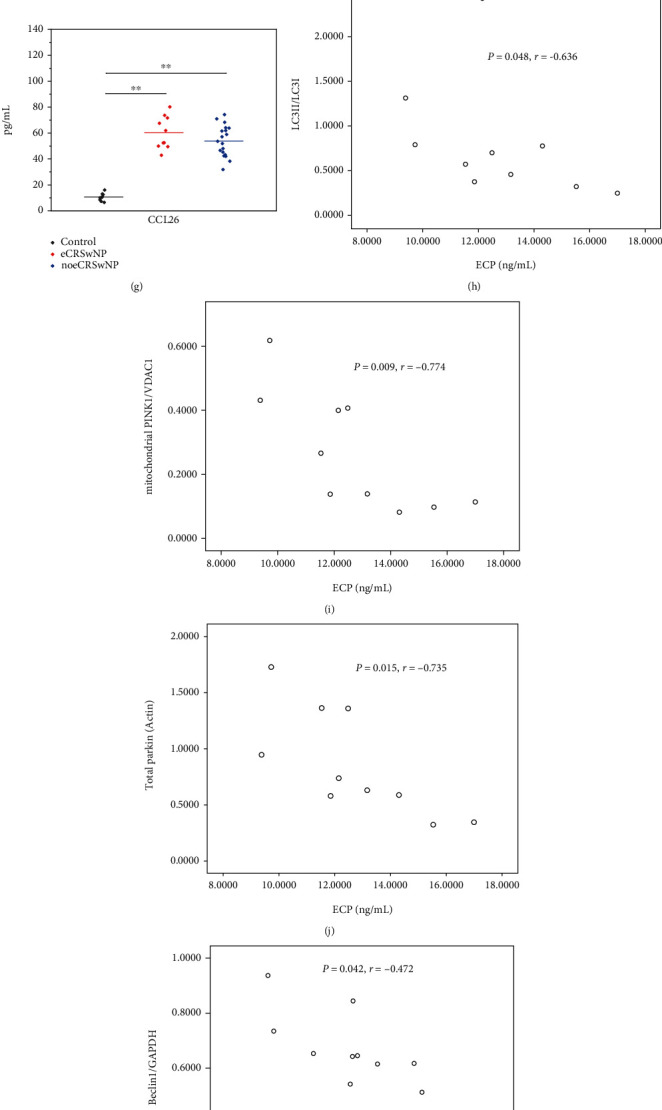
Cytokines in different CRSwNP subtypes. (a) IL-4 levels were increased in patients with eCRSwNP but not in those with noeCRSwNP. (b) IL-5 levels were significantly increased in the nasal polyps of patients with eCRSwNP or noeCRSwNP, compared with control tissues. (c) IL-13 levels were significantly increased in the nasal polyps of patients with eCRSwNP or noeCRSwNP, compared with control tissues. (d) ECP was upregulated in the nasal polyps of patients with eCRSwNP or noeCRSwNP, and particularly high in eCRSwNP, compared with control tissues. (e–g) Levels of the eotaxins CCL11, CCL24, and CCL26 were elevated in the nasal polyps of patients with eCRSwNP or noeCRSwNP, compared with control tissues. (h–j) In patients with eCRSwNP, LC3II/LC3I, mitochondrial PINK1, and total parkin protein levels were significantly inversely correlated with ECP levels. (k) In patients with noeCRSwNP, Beclin 1 protein levels were inversely correlated with ECP levels. (l, m) In patients with noeCRSwNP, LC3II/LC3I and mitochondrial PINK1 protein levels were inversely correlated with CCL11 levels. ∗*P* < 0.05, ∗∗*P* < 0.001.

Next, we investigated the severity of eosinophilic inflammation by measuring cytokine levels. We found that IL-4 levels were increased in patients with eCRSwNP (*P* = 0.028) but not in those with noeCRSwNP (*P* = 0.105; Figure 5(a)). IL-5 and IL-13 levels were significantly increased in the nasal polyps of patients with eCRSwNP (both *P* < 0.001) or noeCRSwNP (both *P* < 0.001), compared with control tissues (Figures 5(b) and 5(c)). ECP was also upregulated in the nasal polyps of patients with eCRSwNP (*P* < 0.001) or noeCRSwNP (*P* < 0.001), and ECP levels were particularly high in patients with eCRSwNP (*P* = 0.045; Figure 5(d)), compared with control tissues. Similarly, levels of the eotaxins CCL11, CCL24, and CCL26 were elevated in the nasal polyps of patients with eCRSwNP (all *P* < 0.001) or noeCRSwNP (all *P* < 0.001), compared with control tissues (Figures 5(e)–5(g)). No significant differences in IL-4, IL-5, IL-13, or eotaxin levels were observed between patients with eCRSwNP and those with noeCRSwNP.

We found no correlations between the levels of cytokines and those of Akt/mTOR pathway proteins in patients with eCRSwNP or noeCRSwNP. We further investigated potential associations between the levels of cytokines and autophagy or mitophagy. In patients with eCRSwNP, LC3II/LC3I (*P* = 0.048, *r* = −0.636), mitochondrial PINK1 (*P* = 0.009, *r* = −0.774), and total parkin (*P* = 0.015, *r* = −0.735) protein levels were significantly inversely correlated with ECP levels (Figures 5(h)–5(j)). In patients with noeCRSwNP, Beclin 1 (*P* = 0.042, *r* = −0.472) protein levels were inversely correlated with ECP levels (Figure 5(k)), whereas LC3II/LC3I (*P* = 0.031, *r* = −0.495) and mitochondrial PINK1 (*P* = 0.047, *r* = −0.461) protein levels were inversely correlated with CCL11 levels (Figures 5(l) and 5(m)).

Together, these data suggest that eosinophilic inflammation increases in patients with eCRSwNP or noeCRSwNP. In addition, autophagy and mitophagy are downregulated in patients with severe eosinophilic inflammation.

### 3.6. Tissue Remodeling in Different CRSwNP Subtypes

**Figure 6:**
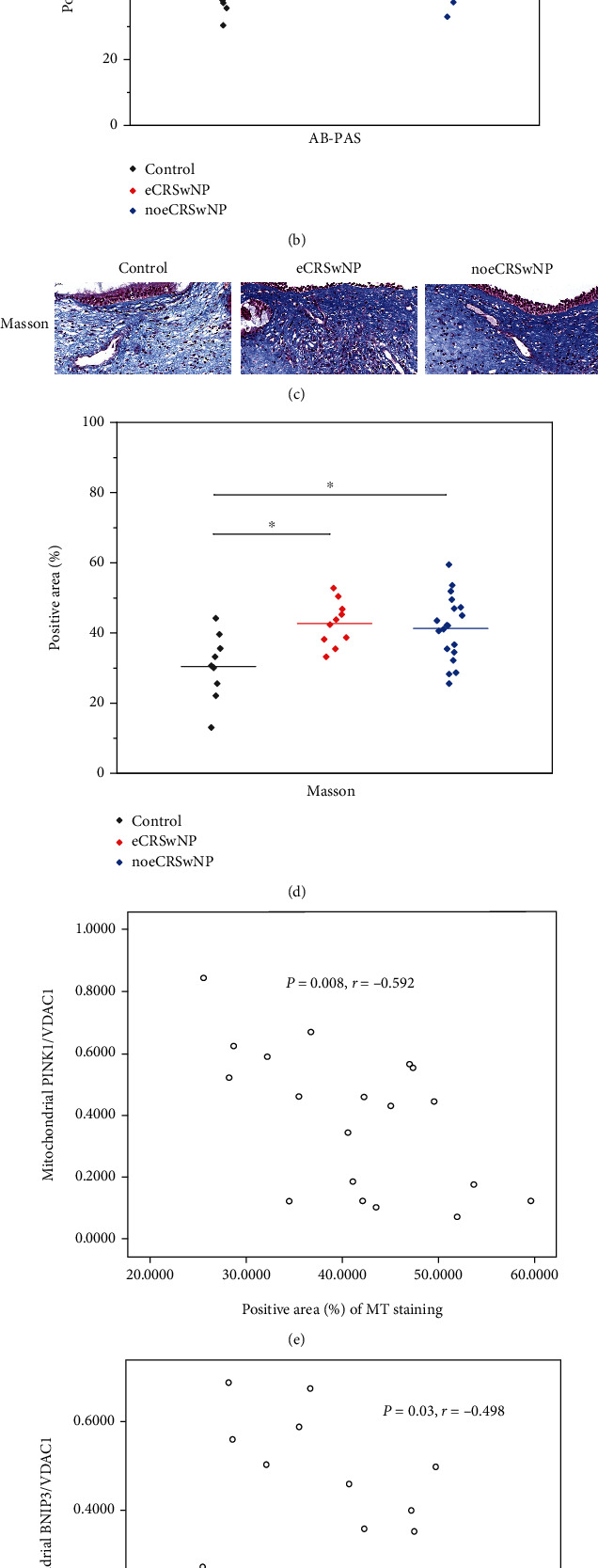
Tissue remodeling in different CRSwNP subtypes. (a, b) PAS–AB stain covered a greater area in sections of the nasal polyps of patients with eCRSwNP or noeCRSwNP, compared with control tissues. (c, d) MT stain covered a greater area in tissue sections from patients with eCRSwNP or noeCRSwNP, compared with control tissues. (e, f) Mitochondrial PINK1 and BNIP3 levels were significantly inversely correlated with the results of MT staining in patients with noeCRSwNP. ∗*P* < 0.05, ∗∗*P* < 0.001.

We investigated tissue remodeling using MT and PAS–AB staining. The PAS–AB stain, which highlights neutral and eosinophilic mucus, covered a greater area in sections of the nasal polyps of patients with eCRSwNP (*P* = 0.002) or noeCRSwNP (*P* = 0.012), compared with control tissues (Figures 6(a) and 6(b)). The MT stain, which highlights the presence of collagen, covered a greater area in tissue sections from patients with eCRSwNP (*P* = 0.004) or noeCRSwNP (*P* = 0.011; Figures 6(c) and 6(d)). We found no correlations between tissue remodeling and the levels of Akt/mTOR pathway- or autophagy-related proteins. Interestingly, mitochondrial PINK1 (*P* = 0.008, *r* = −0.592) and BNIP3 (*P* = 0.03, *r* = −0.498) levels were significantly inversely correlated with the results of MT staining in patients with noeCRSwNP (Figures 6(e) and 6(f)).

These results demonstrate that tissue remodeling is enhanced in patients with eCRSwNP or noeCRSwNP, compared with control tissues. Mitophagy may be downregulated in tissues undergoing extensive remodeling in patients with noeCRSwNP.

## 4. Discussion

Eosinophilic inflammation is a key feature of CRSwNP, particularly eCRSwNP. It is associated with increased disease severity and poor surgical outcomes in patients with CRSwNP [30]. The most common manifestations of eosinophilic inflammation are the infiltration of EOSs and the production of T helper type 2-related cytokines, ECP, and eotaxin. In this study, we found that IL-5, IL-13, ECP, and eotaxin levels were significantly increased in the nasal polyps of patients with eCRSwNP or noeCRSwNP, whereas IL-4 levels were only increased in patients with eCRSwNP. In addition, ECP levels were higher in the nasal polyps of patients with eCRSwNP than in those of patients with noeCRSwNP. These findings show that eosinophilic inflammation plays an important role in both eCRSwNP and noeCRSwNP pathogenesis, while a more enhanced eosinophilic inflammation exists in eCRSwNP subtype.

Abnormal remodeling of the sinonasal mucosa is another feature of CRSwNP [3]. This is characterized by epithelial damage, macrophage and lymphocyte migration, angiogenesis, basement membrane thickening, fibrosis, and edema [31]. Our MT and PAS–AB staining experiments showed that tissue remodeling was upregulated in the nasal polyps of patients with eCRSwNP or noeCRSwNP. These observations are consistent with the results from previous studies [32, 33].

Abnormal autophagy may play an important role in several respiratory diseases. The inhibition of autophagy may decrease cell survival, affecting eosinophilic inflammation in patients with severe asthma [34]. The inhibition of autophagy may also enhance IL-10 production, decreasing inflammation in patients with asthma [35]. One study reported that autophagy released EOS extracellular traps and induced allergic airway inflammation in a murine asthma model [12]. However, the role of autophagy in chronic rhinosinusitis (CRS) remains controversial. Some studies have suggested that autophagy is upregulated in patients with CRS. The levels of LC3II, Beclin 1, and hypoxia-inducible factor-1*α* are elevated in nasal polyps [13]. TEM studies have shown that autophagosomes are formed more frequently in the nasal mucosa and epithelial cells of patients with eCRSwNP or noeCRSwNP compared to control samples [24]. ATG3 expression gradually increased in patients with CRS without nasal polyps, patients with CRSwNP, and mice with CRSwNP/asthma, suggesting a strong link between autophagy and the development of CRS [36]. However, other studies have suggested that autophagy is downregulated in patients with CRS, with LC3 levels reportedly decreased in nasal polyps [25, 26]. The expression of Beclin 1 decreased, whereas p62 mRNA and protein levels increased in nasal polyps, compared with normal inferior turbinate mucosa [37]. In this study, we found that LC3 and Beclin 1 expression levels were lower in patients with eCRSwNP or noeCRSwNP, which is consistent with previous studies [25, 26] and our eCRSwNP mouse experiment (data not shown). Interestingly, significant differences in LC3II/LC3I and p62 protein levels were only found in patients with eCRSwNP. Therefore, deficiencies in autophagy may be particularly important during the onset of eCRSwNP.

Autophagy may regulate eosinophilic inflammation and tissue remodeling by modulating fibroblast and EOS apoptosis, EOS differentiation, and secretion [11]. In patients with severe allergic asthma, autophagy is associated with eosinophilic inflammation [38]. However, autophagy may play different roles, depending on the cell type and disease model used [39]. In an eosinophilic CRS mouse model, knockout of ATG7 in macrophages enhances EOS infiltration, epithelial cell proliferation, and mucosal hypertrophy and exacerbates eosinophilic CRS [15]. We showed that the expression of some autophagy-related proteins was negatively correlated with ECP and eotaxin levels in patients with eCRSwNP or noeCRSwNP. These observations are consistent with the results from previous studies [15]. In murine asthma models, the autophagy inhibitor chloroquine can downregulate airway remodeling [40]. In patients with CRSwNP, hypoxia may stimulate autophagy in fibroblasts, activating tissue remodeling and forming nasal polyps [13]. Nevertheless, we found no correlations between the levels of autophagy-related proteins and the extent of tissue remodeling.

Mitophagy is a form of selective autophagy that degrades damaged or dysfunctional mitochondria under hypoxic or depolarizing conditions or in response to bacterial or viral infections. Abnormal mitophagy may aggravate airway inflammation. The overexpression of mitophagy-related proteins can prevent the accumulation of ROS and cellular senescence, thereby reducing the severity of chronic obstructive pulmonary disease [41]. In patients with acute respiratory distress syndrome, mitophagy may inhibit mitochondria-induced apoptosis, thus alleviating respiratory symptoms [42]. However, the effects of mitophagy remain unclear. Another study suggested that the stimulation of mitophagy may aggravate chronic obstructive pulmonary disease [43]. Recent studies have suggested that the stimulation of mitophagy may influence symptoms in patients with asthma. BNIP3 expression is upregulated in the airway smooth muscle cells of patients with asthma [44]. Another report claims that by suppressing mitophagy in human bronchial epithelial cells, allergic airway inflammation can be attenuated in patients with asthma [45]. PINK1, parkin, and other mitophagy-related proteins are upregulated in fibroblasts from patients with asthma [46]. By contrast, the role of mitophagy in nasal inflammation remains poorly understood. Phosphatase and tensin homolog can prevent nasal inflammation by inhibiting mitophagy in nasal epithelial cells [47]. Bleomycin-A5 can inhibit dynamin-related protein 1-mediated mitophagy in fibroblasts and induce apoptosis in nasal polyps [48]. In this study, we demonstrated that the expression of PINK1, parkin, BNIP3, and FUNDC1 proteins was markedly decreased in the nasal polyps of patients with eCRSwNP, compared with control tissues. The expression of PINK1, BNIP3, and FUNDC1 proteins was also decreased in the nasal polyps of patients with noeCRSwNP. When combined with the TEM observations, our results show that mitophagy is downregulated in patients with eCRSwNP or noeCRSwNP. We believe that in patients with CRSwNP, mitophagy may depend on the PINK1/parkin pathway and the mitophagy receptor proteins BNIP3 and FUNDC1. Moreover, we found that the levels of PINK1/parkin pathway proteins were negatively correlated with ECP and eotaxin levels in patients with eCRSwNP or noeCRSwNP.

Mitophagy regulates fibroblasts to promote airway remodeling [17, 46]. IL-17 may stimulate PINK1-associated mitophagy, accelerating fibrosis in bronchial fibroblasts [28]. Interestingly, we found that mitochondrial PINK1 and BNIP3 levels were negatively correlated with collagen deposition in patients with noeCRSwNP. These results suggest that mitophagy may play an important role in tissue remodeling in patients with noeCRSwNP.

The PI3K/Akt/mTOR pathway operates in various cells and regulates metabolic processes, inflammation, cell survival, and cell division [49]. It plays an important regulatory role in cancer progression and the development of autoimmune diseases and inflammation. In nasal polyps, the levels of PI3K/mTOR proteins are upregulated, and autophagy is downregulated [22]. One study has suggested that the Akt/mTOR pathway is activated in nasal polyp fibroblasts [25]. The Akt signaling pathway is also activated in nasal polyps [21]. In agreement with these findings, we have shown that p-Akt/Akt and p-mTOR/mTOR levels were upregulated in patients with eCRSwNP or noeCRSwNP, demonstrating that the Akt/mTOR pathway was activated. We found no correlation between activation of the Akt/mTOR pathway and autophagy or mitophagy. Recent studies indicate that inhibition of the Akt/mTOR pathway can impair EOS chemotaxis and recruitment, decrease EOS longevity and responsiveness, and attenuate eosinophilic inflammation [50, 51]. Inhibition of the Akt/mTOR pathway can also downregulate tissue remodeling during allergic airway inflammation [52]. However, we found no correlation between activation of the Akt/mTOR pathway and eosinophilic inflammation or tissue remodeling. Further studies using agonists or antagonists of the Akt/mTOR pathway may help determine whether other factors are important.

## 5. Conclusions

This study showed that the Akt/mTOR pathway, eosinophilic inflammation, and tissue remodeling are activated in the nasal polyps of patients with eCRSwNP or noeCRSwNP. The downregulation of autophagy and mitophagy was also observed in eosinophilic and noneosinophilic nasal polyps, and this was negatively correlated with the severity of eosinophilic inflammation. In patients with noeCRSwNP, mitophagy was negatively correlated with the extent of tissue remodeling. The targeting of mitophagy may provide new therapeutic options for different endotypes of CRSwNP.

## Data Availability

The datasets used and/or analyzed during the current study are available from the corresponding author on reasonable request.
